# Clinical evidence of photobiomodulation therapy (PBMT) on implant stability and success: a systematic review and meta-analysis

**DOI:** 10.1186/s12903-019-0779-4

**Published:** 2019-05-07

**Authors:** Yuan Chen, Caojie Liu, Xinlei Chen, Anchun Mo

**Affiliations:** Department of Oral Implantology, West China Hospital of Stomatology, Sichuan University, Chengdu, 610041 China

**Keywords:** Dental implant, Implant stability, Implant success rate, Low level laser therapy, Photobiomodulation therapy

## Abstract

**Background:**

Photobiomodulation therapy (PBMT), a type of light therapy that uses the concept of photobiomodulation, is developed to promote bone healing. Clinical studies have been conducted to assess the influence of PBMT on dental implant stability and success rate. This is the first systematic review and meta-analysis to assess the effect of PBMT and methodological quality of these studies on implants in human clinical trials.

**Methods:**

An electronic search was performed in Pubmed, Embase, and the Cochrane Controlled Register of Trials (CENTRAL).

**Results:**

Initially, 675 articles were identified, among which only 8 met the inclusion criteria. Four of the 8 studies presented a low risk of bias, whereas the other 4 were of moderate risk. Our review focused on implant success rates and implant stability measured at days 0 and 10, and at 3, 4, 6, and 12 weeks. No significant differences were observed between the PBMT group and the control group regarding implant stability or success rate.

**Conclusions:**

The existing clinical studies did not provide sufficient evidence to observe positive effects of PBMT on implants in patients. An increased number of high-quality clinical randomized controlled trials (RCTs) are required to verify the data and to draw convincing conclusions.

## Background

Because of its excellent aesthetics, functional characteristics, and high success rates, implantology has been increasingly popular among patients with dentition defects [1]. However, there is still a risk of failure due to a number of complex factors, with lack of osseointegration as the main reason [2].

Osseointegration is a core definition in implantology, and describes the direct structural and functional connection between live bone and the surface of an implant [3]. Throughout osseointegration, osteoblasts and osteoclasts interact and influence each other. Osseointegration is considered one of the most important determinants of implant stability [4], and is a key factor that determines success of the implant. However, a number of factors can influence osseointegration [5]. Therefore, increased attention has been paid to the physical, chemical, and biological attempts to promote osseointegration, one of which is photobiomodulation therapy (PBMT) [6].

PBMT, also known as low level therapy (LLLT), is defined as ‘a type of noninvasive and nonthermal therapy based on non-ionizing light sources, including lasers, light-emitting diodes (LEDs), and broadband light, in the visible and infrared spectrum’ [7]. In the 1990s, the Food and Drug Administration (FDA) approved laser therapy for oral treatment, therefore its application in surgery and endodontic treatment has been among the most popular topical treatments, e.g. treatment of mucosal leukoplakia, pediatric dental diseases, and alveolar osteitis [8]. Laser classification is complex and involves a large variety in different categories, such as excitation source, wavelength, and intensity. In medical applications, the power of the laser is an important parameter for therapy [9]. LED is an alternative for the laser because their effects on tissue are similar, and LED was given FDA approval [10].

In previous studies, the clinical effect of PBMT has been well characterized, e.g. it alleviates inflammation and pain, and promotes wound healing [11]*.* The bio-stimulating effect of PBMT has aroused the interest for research although there a clear mechanism of action is lacking [12]. Many studies have shown that PBMT contributes to bone healing [13, 14], and close attention has been given to the bio-stimulating effects of PBMT on osteoblast proliferation and osteogenesis after implant therapy [15]. Moreover, in previous studies it has been attempted to establish the most appropriate wavelength, dose, frequency, etc. to provide a protocol for the use of PBMT in implantology [16].

To ascertain the effect of PBMT on improving implant stability, previous studies have focused on animals, including rodents, rabbits, beagles, and primates*.* [2]. A number of studies and systematic reviews have been conducted, and suggested that PBMT provided a positive effect in animal models [1, 2, 17]. However, due to the lack of clinical data, these studies could not provide powerful evidence for a positive effect of PBMT in humans. Fortunately, a number of clinical trials have recently been published, thereby increasing the cohort of treated patients. Thus, in this systematic review and meta-analysis, we aimed to evaluate the clinical effects of PBMT on implant stability and success in humans.

## Methods

This systematic review and meta-analysis was performed according to the Preferred Reporting Items for Systematic Reviews and Meta-Analyses (PRISMA) guidelines [18].

### Focused question

It has been previously been established that PBMT promotes the osseointegration process in animals, therefore, our focused question was addressed based on the Participants, Interventions, Control and Outcomes (PICO) principle: ‘For patients receiving implant treatment, does PBMT enhance implant stability and success rate?’

### Search strategy

In this study, a literature search of the databases Pubmed, Embase, and the Cochrane Controlled Register of Trials (CENTRAL) was conducted up to November 2018. For each database Appropriate search algorithms were developed using the following terms: (laser OR laser therapy OR laser irradiation OR phototherapy OR low-level laser OR low-intensity laser OR low-output laser OR soft laser) AND (implant) AND (stability OR osseointegration). The search was limited to human subjects and without restriction in publication language.

Two blinded, independent investigators screened the titles and abstracts identified by the electronic search to select potentially eligible studies. Subsequently, the full text of the candidate studies was further evaluated to identify studies that met all inclusion criteria. To avoid missing any eligible studies, the references of all included articles and relevant reviews were also searched. In addition, a manual search was performed using the following dental implant and laser journals: Lasers in Medical Science; The International Journal of Oral and Maxillofacial Implants; The Journal of the American Dental Association; Journal of Oral and Maxillofacial Surgery; Journal of Periodontology; Photomedicine & Laser Surgery, and The Journal of Prosthetic Dentistry. Agreement between reviewers in the selection procedure was calculated by the Cohen’s kappa statistics, assuming κ = 0.6 as an eligible score [19]. Any discrepancies were resolved by discussion or consultation with a third reviewer.

### Inclusion and exclusion criteria

This review only focused on randomized controlled trials (RCTs) or quasi-RCTs reporting the effects of PBMT on implant stability and/or implant success rate in human clinical trials. Case reports, review papers, letters to the editor, monographs, in vitro studies, animal studies, and studies recruiting patients with systemic diseases or those being medically treated were excluded.

### Data collection and analysis

The following information was extracted from the studies included in this review by two independent reviewers: first author (year), country, sample size, mean age (range), study type, the position of implant, type of implant and surface, bone condition, analysis performed, evaluation time, type of PBMT, wavelength, mode, output/energy (density), total dose per point (implant), exposure time, and frequency of laser treatment. The corresponding author was contacted to obtain any incomplete or missing data. Disagreements were resolved by discussion, with arbitration by a third reviewer, if necessary.

Statistical analysis was performed using RevMan 5.3 software [20] provided by the Cochrane Collaboration. For implant stability, the mean differences (MD), and 95% confidence intervals (CI) were calculated. Implant success rate was classified as dichotomous data, thus the effect of intervention was estimated as a risk ratio (RR) with a 95% CI. The I^2^ statistic was used to evaluate trial heterogeneity using α = 0.10. A random-effects model was employed to analyze data exhibiting substantial heterogeneity (I^2^ > 50%). In other cases, a fixed-effects model was used. Statistical significance was set at α < 0.05 (two-tailed z tests). If a meta-analysis could not be performed, data were summarized qualitatively.

### Quality assessment

The quality of all the studies included in the review was assessed by two investigators who were blinded, using the Cochrane risk of bias assessment tool [21]. The agreement between reviewers was assessed based on Cohen’s kappa statistics, assuming κ = 0.6 to be an eligible score [19]. Any disagreement was resolved by discussion, and a third reviewer was consulted if arbitration was required. The assessment of all articles encompassed seven domains: random sequence generation, allocation concealment, blinding of participants and outcome assessors, incomplete outcome data, selective reporting, and other bias. Each domain was divided into low risk of bias, unclear risk of bias, or high risk of bias. A study was regarded low risk only if all domains were evaluated as low risk. If one or more domains were evaluated as unclear, the study was categorized as being of moderate risk. Any domain examined as high risk resulted in that study being classified as high risk of bias.

## Results

### Study characteristics

**Fig. 1:**
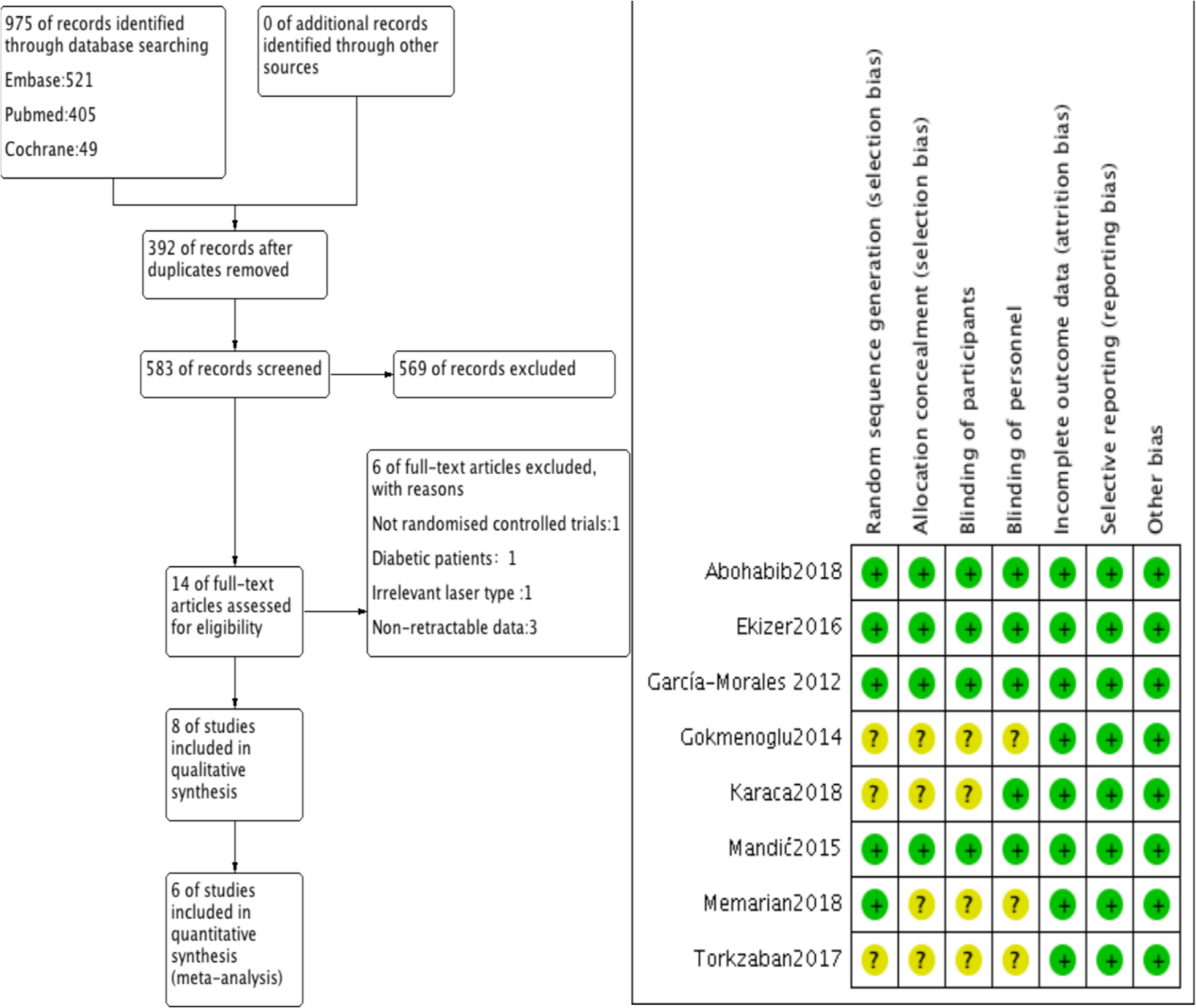
Flowchart depicting the study selection process and risk of bias summary

During the search process, a total of 975 articles were identified (Fig. 1). After screening the titles and abstracts, the full texts of 14 articles were obtained, and further evaluated by two independent investigators (inter-reviewer agreement, kappa = 0.92). Finally, 8 studies were selected, of which 6 were chosen for meta-analysis [22–29].

Six out of the 8 studies were published after 2015. Of these, 3 studies were conducted in Turkey, 2 in Iran, and the remaining 3 were conducted in Egypt, Serbia, and Brazil. In 7 studies, the mean age or age range was described, ranging from 13.1 to 75 years old. The timing of examinations varied among studies, and involved mostly 0 and 10 days, and 3, 4, 6, 12 weeks after dental implant insertion. The parameters of PBMT also varied among studies. Five used low level lasers with a continuous wave, 2 used LED, and 1 used a low level laser or LED. The wavelength of the PBMT varied from 618 nm to 940 nm with power output ranging from 20 mW to 1700 mW. Further characteristics of the studies are summarized in Table 1, the parameters of the lasers are presented in Table 2.

**Table 1:** Characteristics of included studies

Author(year)	Country	Sample size	Mean age (range)	Study type	The position of implant	Type of implant and surface	Bone condition	Performed analysis	Evaluation time
Abohabib(2018)	Egypt	15	20.9 ± 3.4	Split mouth	Between the maxillary second premolar and first molar buccally	AbsoAnchor orthodontic mini-implants (AbsoAnchor, Dentos, Daegu, Korea) with a diameter of 1.5 mm and a length of 8 mm	NR	RFA in Hertz	Immediately and 1, 2, 3, 4, 6, 8, 10 weeks later
Mandić(2015)	Serbia	12	61.28(55–75)	Split mouth	Premolar and/or molar maxillary regions	Self-tapping BlueSky® implants(Bredent, Germany) with a diameter of 4 mm and a length of 10 mm	Type D3 and D4	RFA in ISQ	Immediately and 1, 2, 3, 4, 5, 6 weeks later
Karaca(2018)	Turkey	25	51.2 ± 2.3(36–64)	NR	Posterior mandible	DTI Implant Systems(Istanbul, Turkey) with a diameter of 4 or 4.5 mm and a length of 10 mm	Minimum 12 mm above the mandibular canal and minimum 5.0 mm in width	RFA in ISQ	Immediately and 6 months later
García-Morales (2012)	Brazil	8	36(20–55)	Split mouth	Posterior mandible	XiVE-S implants(Dentsply Friadent, Mannheim, Germany) with a diameter of 3.8 mm and a length of 11 mm	Type 2	RFA in ISQ	Immediately,10 days and 3,6,9,12 weeks later
Memarian(2018)	Iran	12	NR	Split mouth	Mandible(one in the midline and the other two at the left and right canine teeth positions)	DIO implants (Korea) with resorbable blast media surface(invasive fungal infections-tissue level)	Type 2 or 3	PTV	Immediately and 3,4,8 weeks later
Ekizer(2016)	Turkey	20	16.77 ± 1.41 (13.1–19)	Split mouth	Between the roots of maxillary first molars and second premolars	Screw-shaped titanium orthodontic miniscrews with a diameter of 1.6 mm and a length of 8 mm	NR	RFA in ISQ	Immediately and 1,2,3 months later
Gokmenoglu (2014)	Turkey	15	LED:50.43 ± 9.25; C:45.87 ± 13.46	NR	NR	XiVE implants(Dentsply-Friadent, Mannheim, Germany) with a diameter of 3.8 (3.5–4.5)mm and a length of 11.0(11–11)mm in LED group, while with a diameter of 4.5((3.8–4.5)mm and a length of 11.0 (10.6–11.0)mm in control group	Type 2 or 3	RFA in ISQ	Immediately and 2,4,8,12 weeks later
Torkzaban(2017)	Iran	19	Female:43 Male:40.8	NR	Maxillary teeth	Dio implants (Dio UF, Busan, Korea) with a diameter of 4 or 4.5 mm a length of 10 or 11.5 mm	Type D3 and D4	RFA in ISQ	Immediately,10 days and 3,6,12 weeks later

*NR* Not reported, *RFA* Resonant frequency analysis, *PTV* Periotest value, *ISQ* Implant stability quotient, *C* Control group

**Table 2:** Parameters of PBMT in the included studies

Author(year)	Type of LLLT	Wavelength	mode	Output/energy(density)	Total dose Per point(implant)	Exposure time	Frequency of laser treatment
Abohabib(2018)	Biolase diode laser (Epic 10 Console)	940 nm	Continuous wave	1.7w;36 J/cm2	102 J/implant	60s	Immediately after implant insertion and 7,14,21 days later
Mandić(2015)	GaAlAs laser(Medicolaser 637, Technoline, Belgrade, Serbia)	637 nm	Continuous wave	40mw;6.26 J/cm2	NR	NR	Immediately after implant insertion and 1,2,3,4,5,6,7 days later
Karaca(2018)	GaAlAs laser(Laser BTL-4000, Brno, Czech Republic)	830 nm	Continuous wave	86 ± 2 mW;92.1 J/cm2	0.25 J/point; 5 J/per teeth	60s	Repeated every two days for 2 weeks
García-Morales(2012)	GaAlAs laser(Thera Lase, DMC, São Carlos - SP, Brazil)	830 nm	Continuous wave	86 ± 2 mW;92.1 J/cm2	0.25 J/point; 5 J/per implant	60s	Repeated every two days for 2 weeks
Memarian(2018)	Diode laser(Diode laser doctor smile 810 nm, Italy); LED(Osseopulse™AR 300, Biolux Research Ltd., Vancouver, British Columbia, Canada)	Laser:810 nm LED:626 nm	Laser: Continuous wave LED:NR	Laser:50 mW;20 J/cm2. LED:185 mW;46.2 J/cm2	Laser:20 J/implant LED:222 J/implant	Laser:400 s LED:1200s	Immediately after implant insertion and 3,7,10,14 days later
Ekizer(2016)	LED(Osseopulse™ Biolux Research Ltd., Vancouver, Canada)	618 nm	NR	20 mW/cm2	NR	1200s	Once a day during 21 days
Gokmenoglu(2014)	LED(Osseopulse™ AR 300, Biolux Research Ltd., Vancouver, British Columbia, Canada)	626 nm	NR	185 mW.46.2 J/cm2	222 J/implant	1200s	Three times per week for 3 weeks
Torkzaban(2017)	Biolase diode laser(epic10, BIOLASE, Inc., Irvine, CA, USA)	940 nm	Continuous wave	100 mW;28.37 J/cm2	8 J/implant	80s	Repeated at 2, 4, 6, 8, 10, and 12 days after imlant insertion

*NR* Not reported, *GaAlAs* Gallium-aluminum-arsenide

### Quality analysis

The risk of bias in the studies included in the review was assessed according to the Cochrane Handbook (Fig. 1). Four studies presented low risk of bias [24, 26, 27, 29], whereas the other 4 were of moderate risk [22, 23, 25, 28]. Unclear information about ‘random sequence generation’, ‘blinding of participants’, ‘blinding of personnel’ and ‘allocation concealment’ accounted for the risk factors. Of the 4 studies with moderate risk, the blinding to participants or allocation concealment was not mentioned. Furthermore, in 3 studies, blinding of personnel was not mentioned, and in 3 studies, the specific randomization methods were not mentioned. The agreement between the reviewers was 0.91.

### Primary outcomes-the effect on implant stability

Eight studies reported the effects of PBMT on implant stability at different follow-up times. Of these, the evaluation periods varied considerably. In our review, we only pooled the outcomes measured at days 0 and 10, and at 3, 4, 6, 12 weeks after implant placement. For data with implant stability quotient (ISQ) measurements, we conducted a meta-analysis, otherwise, the outcomes were only qualitatively described.

#### Outcomes measured immediately and 10 days after implants were placed

**Fig. 2:**
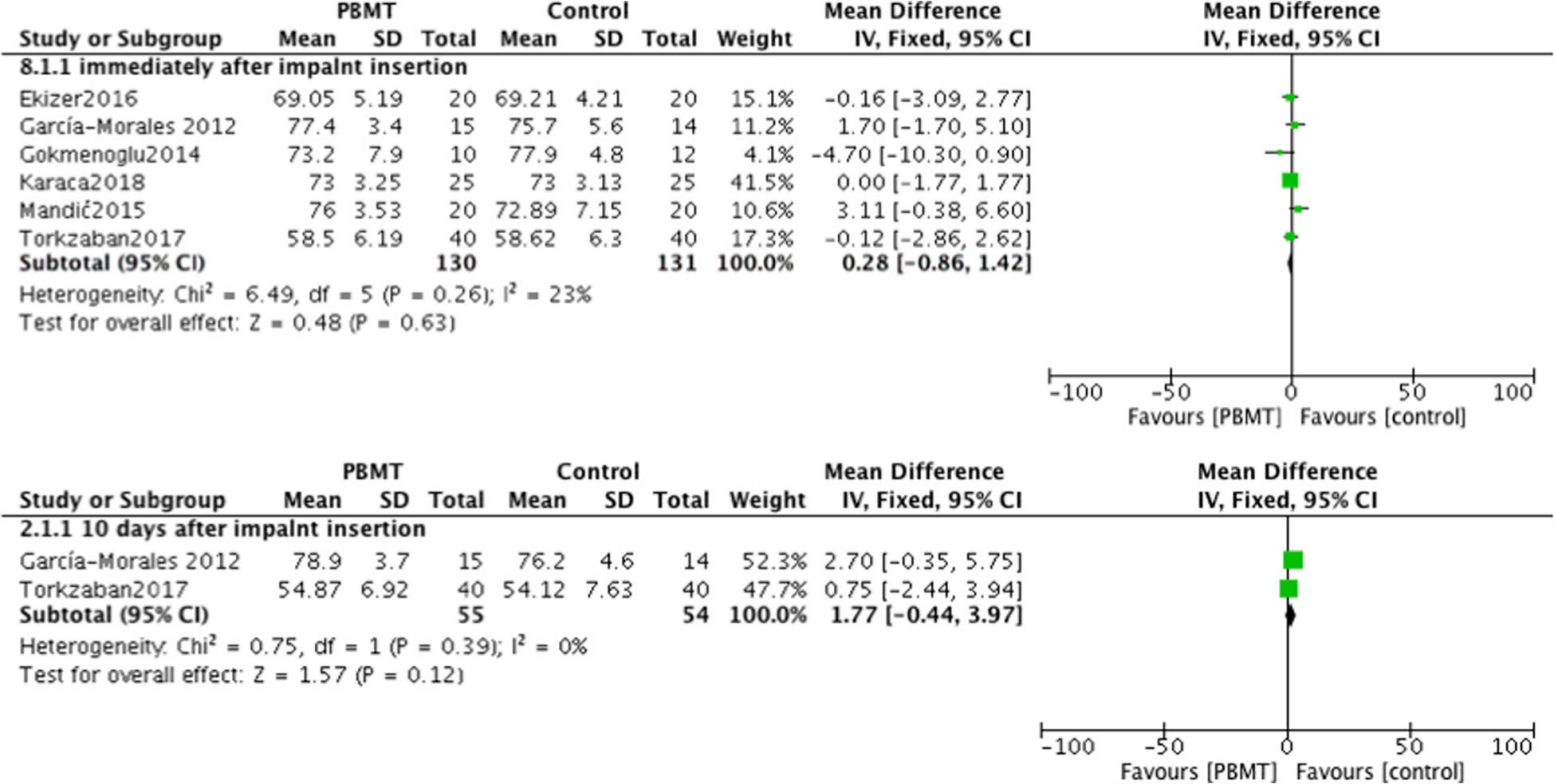
Comparison: Photobiomodulation therapy versus control, outcome: implant stability quotient measured immediately and 10 days after placing of the implant

All 8 studies included in the review assessed implant stability immediately after the implant was placed. Among them, 6 studies used ISQ measurements, thereby enabling synthesis of the data by meta-analysis [22, 23, 26–29]. The results of the meta-analysis verified that no significant differences were observed in implant stability between the PBMT group and the control group (I^2^ = 23%; *P* = 0.63; MD = 0.28; 95% CI: -0.86-1.42) (Fig. 2). One study reported resonance frequency analysis (RFA) values in Hertz and 1 reported Periotest values (PTV), and neither of showed significant differences between the 2 groups measured immediately after implantation [24, 25]. Two of the 8 studies reported implant stability using the ISQ index measured 10 days after implant insertion. The results also demonstrated no significant differences between treatment and control groups (I^2^ = 0%; *P* = 0.12; MD = 1.77; 95% CI: -0.44-3.97) (Fig. 2).

#### Outcomes measured 3 and 4 weeks after placement of implants

**Fig. 3:**
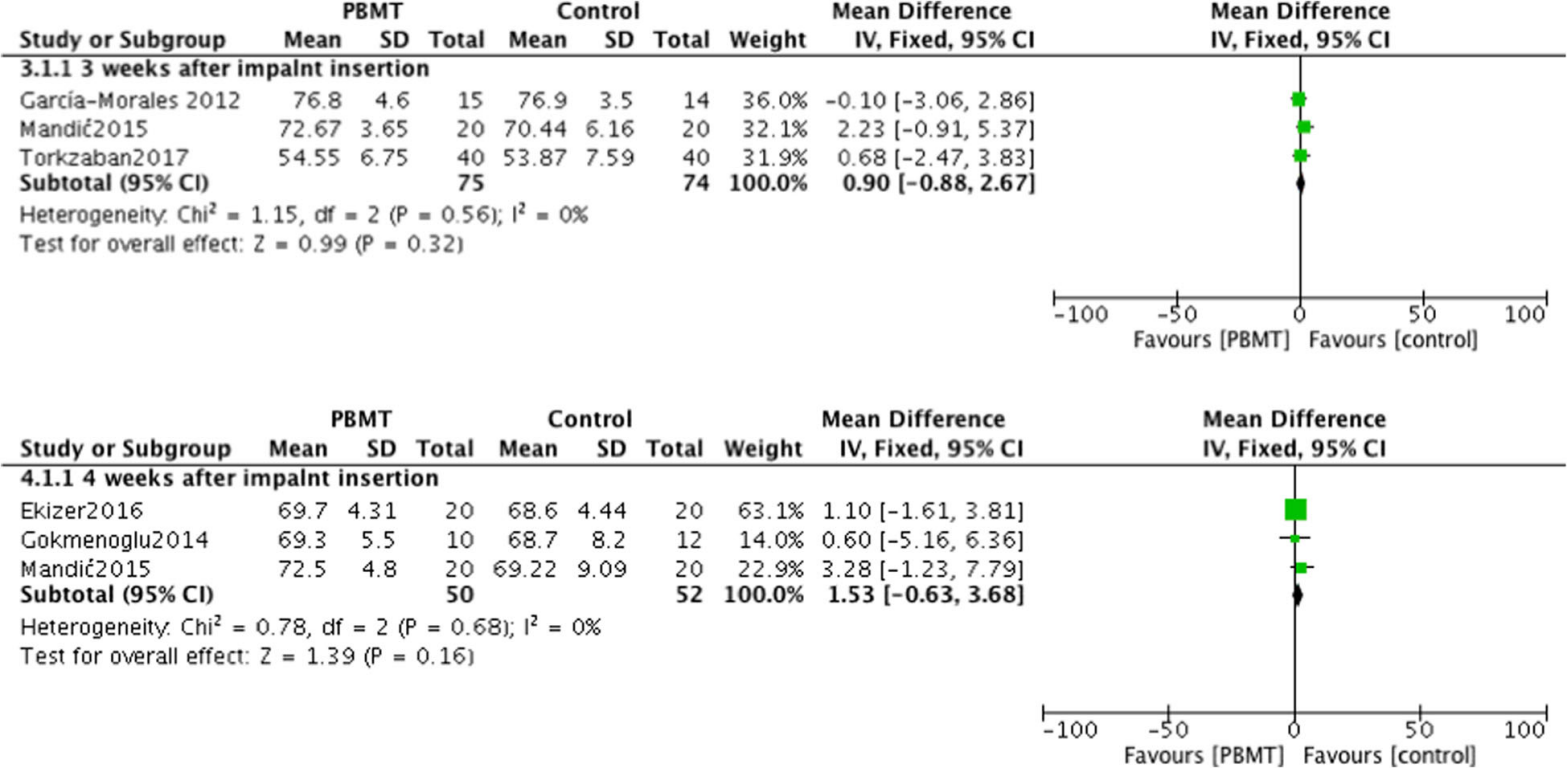
Comparison: Photobiomodulation therapy versus control, outcome: implant stability quotient measured 3 and 4 weeks after placing of the implant

In five studies, implant stability data was provided and was measured 3 weeks after implant placement [23–25, 27, 29]. However, 1 reported RFA in Hertz and 1 reported PTV. Therefore, these 2 studies were not included in the meta-analysis [24, 25]. Although PBMT improved the ISQ index when compared with controls, a mean difference of 0.9 was not statistically significant (I^2^ = 0%; *P* = 0.32; MD = 0.9; 95% CI: -0.88-2.67) (Fig. 3). Five studies reported outcomes 4 weeks after implant insertion [24–28], however those reporting PTV and RFA were excluded from the meta-analysis. No significant differences were observed in the results of the remaining 3 studies (*P* = 0.16), with very small heterogeneity in the data (I^2^ = 0%, *P* = 0.68) (Fig. 3). However, Abohabib et al. measured implant stability using RFA in Hertz and reported significant differences between the PBMT and control groups at 3 and 4 weeks after implant insertion (3 weeks: *P* = 0.032; 4 weeks: *P* = 0.047) [24]. Memarian et al. compared low level laser and LED with a control group [25], and the results indicated significant improvement after LED and low level laser treatment 3 and 4 weeks after implantation.

### Outcomes measured 6 weeks after implant placement

**Fig. 4:**
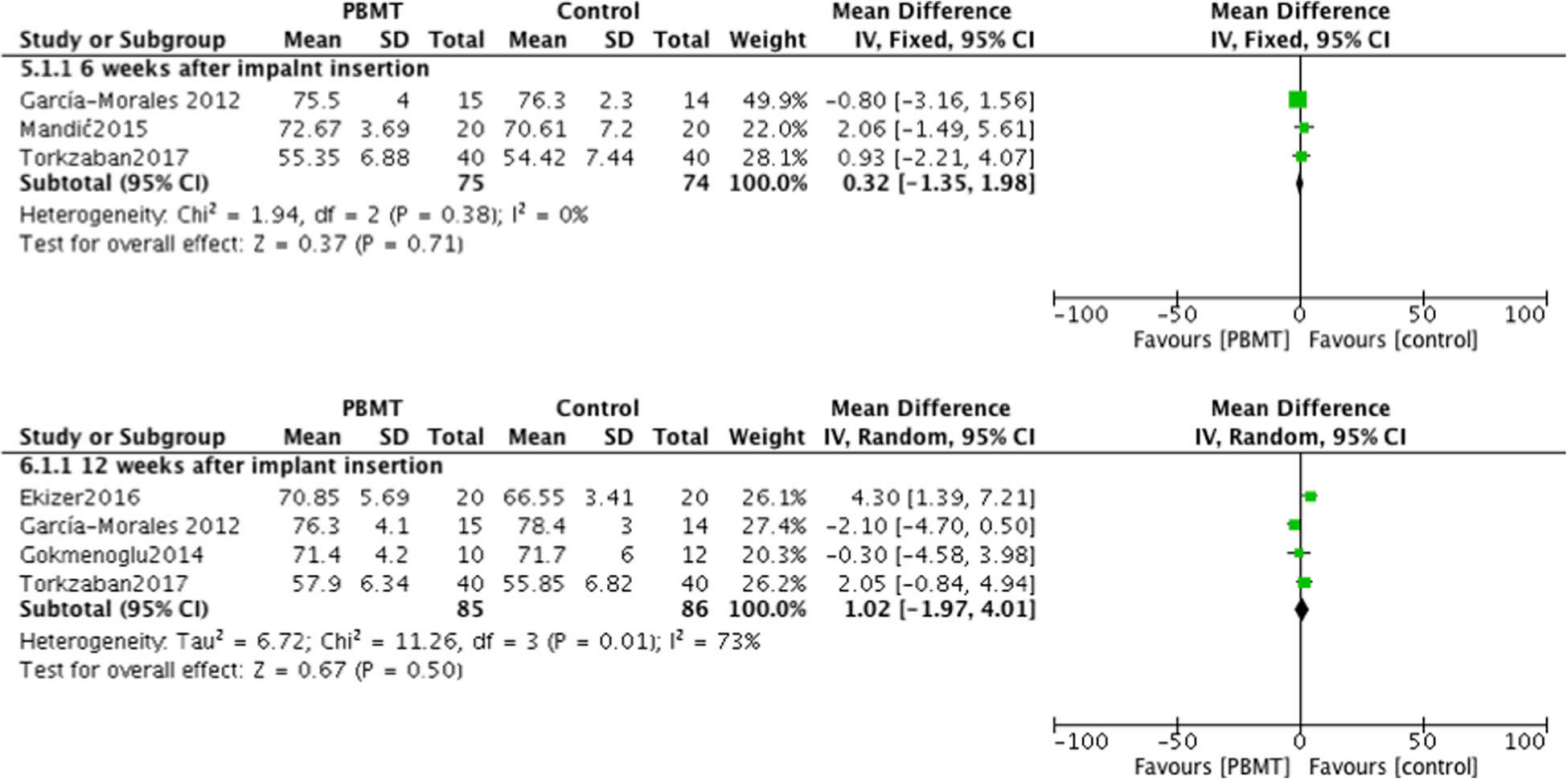
Comparison: Photobiomodulation therapy versus control, outcome: implant stability quotient measured 6 and 12 weeks after placing of the implant

In 4 studies, data concerning implant stability were measured 6 weeks after implant insertion [23, 24, 27, 29]. Abohabib et al. reported RFA in Hertz, demonstrating a significant difference between PBMT and the control group (*P* = 0.016) [24]. In the remaining 3 studies, ISQ measurements were used and were thus included in the meta-analysis. PBMT provided no significant improvement in implant stability (I^2^ = 0%; *P* = 0.71; MD = 0.32; 95% CI: -1.35-1.98) (Fig. 4).

### Outcomes measured 12 weeks after implant placement

In 4 studies, adequate implant stability data measured 12 weeks after placing of the implants were reported [23, 26, 28, 29], on which meta-analysis was conducted. No significant differences were observed between PBMT groups and control groups, with significant heterogeneity in the data (I^2^ = 73%; *P* = 0.50; MD = 1.02; 95% CI: -1.97-4.01) (Fig. 4).

### Secondary outcomes-implant success rate

**Fig. 5:**
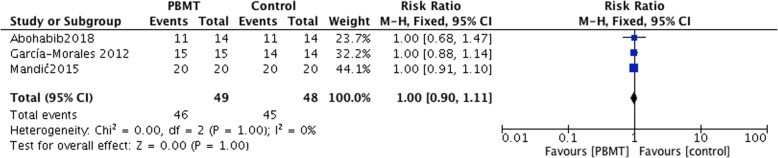
Comparison: Photobiomodulation therapy versus control, outcome: success rate of implants

Four studies reported the success rate of dental implants [22, 24, 27, 29]. In a study by Karaca et al., only the overall success rate of all groups 6 months after implant insertion was reported (92%) [22]. Thus, a meta-analysis was conducted on the remaining 3 studies. A forest plot revealed that the implant success rate of the PBMT group was similar to that of the control group (I^2^ = 0%; *P* = 1; MD = 1; 95% CI: 0.9–1.11) (Fig. 5).

## Discussion

### Quality of the studies

Based on the quality assessment of the study, half of the included studies were considered to have moderate risk of bias. Among them, 2 studies presented unclear information about ‘random sequence generation’, ‘allocation concealment’, ‘blinding of participants’, and the ‘blinding of personnel’, 1 study was vague about ‘random sequence generation’, ‘allocation concealment’, and ‘blinding of participants’, whereas in the other one, the methods of ‘allocation concealment’, ‘blinding of participants’, and ‘blinding of personnel’ were not explicitly described. In general, these methodological drawbacks may lead to bias, thereby affecting the reliability of some results. For example, selection bias can be a result of a tendency to be subjective, and contribute to false positive or negative results [18]. Imperfection of blinding techniques may exaggerate the effect, and result in false positive results [18].

### Effect of PBMT on implant stability and success rate

In this systematic review, 6 studies used RFA in ISQ as their measurement technique for the evaluation of implant stability, for which data were pooled for meta-analysis. However, in the other 2 studies, RFA in Hertz and PTV were used respectively and were only summarized qualitatively. Objective measurements, including RFA or PTV are often used to test implant stability and osseointegration [14]. For the RFA technique, a SmartPeg is inserted on the top of the implant, then the implant resonates with the vibration of the magnetic pulse, and the frequency in Hertz would be recorded [16]. Following computer-aided data analysis, resonant frequency in Hertz is converted into an ISQ index [30]. Higher ISQ values, values varying from 1 to 100, indicate better implant stability and better osseointegration, [2]. On the contrary, a lower PTV often indicates better implant stability with a range of − 8 to 50 [1].

As shown in the meta-analysis, no significant differences were observed regarding implant success rate and implant stability (measured at days 0 and 10, and at 3, 4, 6, 12 weeks) between the PBMT group and control group. As for the other two studies, which were excluded from meta-anaysis, Memarian et al. used PTV and reported significant improvement in implant stability 3 and 4 weeks after treatment with LED or low level laser when compared with control group. Abohabib et al. used RFA in Hertz and also observed a significant increase in implant stability in the PBMT group compared with control group after 3 to 10 weeks. Although in animal studies it was established that PBMT increased bone-implant contact, and improved the production of OPG and RANKL without negative effects on bone resorption [31], existing clinical data did not provide sufficient evidence that PBMT has a positive effect on implant stability or success rate in humans. Herein, we have several hypotheses to explain the insignificant effects of PBMT on humans, which was inconsistent with the data presented in animal studies.

First of all, study design may be a factor. It is well known that a good study design is vital to obtain accurate results. However, the study design in several studies included in this review was not highly satisfactory. For example, several studies were confined to patients with a certain age range or bone condition. Thus, the data were limited and not suitable for the entire population. Additionally, the lack of significant results may be attributed to the limited number of pooled studies and methodological defects, including inadequate countermeasures to avoid foreseeing interventions.

Secondly, the unsuitable treatment protocols of PBMT and high primary stability of implants may be a causal factor. In previous studies, it has been demonstrated that the effect of PBMT is related to the treatment protocol, including wavelength, mode, output, energy density, exposure time, and frequency of treatment [32]. Thus, unsuitable treatment protocols of PBMT may decrease the photobiomodulation efficacy on target areas, such as low energy density which does not reach the optimal therapeutic window. Moreover, most of the included studies used split-mouth design to control the experimental conditions, thus the scattering spreading energy may also affect the control sites. As indicated previously, the effect of PBMT may be masked by high primary stability of implants, which is associated with bone condition, implant surface and underized drilling technique. When the primary stability was high enough, a small change in stiffness may not be found during measuring.

Furthermore, complex human body environments would also influence the effect of PBMT. The baseline of implants in humans is often more complex compared to that in animal models. In clinical practice, patients may have dental defects caused by numerous factors, such as trauma, periodontitis or periapical periodontitis [1], following alveolar resorption, and experience severe inflammation at the site of implantation prior to surgery [31]. These complex pathological environments should be taken into consideration, because the circumstances surrounding an implant could have a significant impact on implantation outcome [32]. Additionally, the different biological nature between animals and humans may result in different biochemical reactions towards PBMT, thereby causing inconsistent treatment outcomes between the two experimental models [33–36]. It is important to note that the ossification mechanisms and function activities of tibia, femur, and human jaw bones are different. Thus, in several previously published animal studies, with implants inserted into the tibia and femur rather than the maxillary or mandibular bone, the placement of implants in jaw bones could not be simulated [37].

### Limitations of this study

This systematic review and meta-analysis have some limitations. Firstly, only 8 studies were included, which was considerabely less than aimed for. Moreover, due to the small sample size, subjects could not be divided into corresponding groups based on age or sex [38]. Such inter-subject variations may result in clinical heterogeneity.

Secondly, baseline charateristics of bone quality in included studies varied significantly, which was reported as good in 4 studies (type 2 or 3 or reporting sufficient quantities of bone) [17–20], poor in 2 studies (type D3 or D4) [11, 22], and not disclosed in the remainder of the studies [21, 23]. Furthermore, several uncontrollable factors were present, including varying dietary habits, oral hygiene status, and awareness of oral health between patients [39]. These differences may result in inconsistent baseline parameters, which further influence collected data and analysis of the results, especially for implant stability [40]. An improved experimental design should take such factors into consideration and publicity and education in oral hygiene measures should be conduced to reduce inflammation around the implants, so as to control the baseline parameters, and acquire more reliable data [18]. Moreover, the implant material applied in all included studies was titanium. Our conclusion was limited due to the simplex impant material, which is more appropriate for titanium implants. However, since zirconia implants have gained increased attention [41], it would also be important to explore the effect of PBMT on zirconia implants.

### Directions for future research

Given the limitations mentioned above, the following strategies were suggested to indicate directions for future research. Firstly, more high-quality RCTs in humans are clearly warranted to verify the data and draw rigorous conclusions, which should be strictly conducted according to the Cochrane’s risk of bias criteria. Secondly, the bone conditions of all patients and PBMT parameters applied in the study should be reported in detail. Thirdly, significant concern should be taken in the effect of PBMT to different implant materials such as zirconia. At last, in order to explore the best ‘therapeutic window’ of PBMT, gradients for correlative PBMT parameters should be set in clinical trails.

## Conclusions

Several animal studies have indicated that PBMT could facilitate hard and soft tissue regeneration, promote osseointegration, and improve implant stability, however, existing clinical studies do not support the satisfactory effect of PBMT on implant stability or the success rate in humans. Due to limitations of current study, additional high-quality human clinical trials are required to verify the data for a more convincing conclusion.

## Abbreviations

CI: Confidence Intervals
FDA: Food and Drug Administration
ISQ: Implant Stability Quotient
LED: Light-emitting diode
LLLT: Low Level Therapy
MD: Mean Differences
PBMT: Photobiomodulation Therapy;
PICO: Participants, Interventions, Control and Outcomes
PRISMA: Preferred Reporting Items for Systematic Reviews and Meta-Analyses
PTV: Periotest Values
RCTs: Randomized Controlled Trials
RFA: Resonance Frequency Analysis
RR: Risk Ratio
